# Synthesis, Characterization, and Thermal Kinetics of Mixed Gadolinium: Calcium Heptamolybdate System

**DOI:** 10.1155/2014/141463

**Published:** 2014-10-20

**Authors:** R. K. Koul, Shivani Suri, Vishal Singh, K. K. Bamzai

**Affiliations:** Crystal Growth & Materials Research Laboratory, Department of Physics and Electronics, University of Jammu, Jammu 180006, India

## Abstract

Synthesis of mixed gadolinium calcium heptamolybdate (GdCaHM) system in silica gel medium using single gel single tube technique has been successfully achieved. The grown crystal exhibits various morphologies, which includes spherulites, multifaceted, and square platelets. The nature of the grown material was established by X-ray diffraction (XRD) studies. Fourier transform infrared spectroscopy (FTIR) study signifies the presence of heptamolybdate (Mo_7_O_24_) and water symmetry structure, whereas energy dispersive X-ray analysis (EDAX) establishes the stoichiometric of the grown crystal as GdCaMo_7_O_24_·8H_2_O. The thermal behaviour was studied using the thermoanalytical techniques, which include thermogravimetry (TG), differential thermal analysis (DTA), and differential scanning calorimetry (DSC). Results obtained on the application of TG based models, namely, Horowitz-Metzger, Coats-Redfern, and Piloyan-Novikova, suggest the contracting cylindrical model as the relevant model for the thermal decomposition of the material. The kinetic parameters, namely, the order of reaction (*n*), activation energy (*E*_*a*_), frequency factor (*Z*), and entropy (Δ*S*∗), were also calculated using these three models.

## 1. Introduction

Rare earth (RE) compounds in general have been recognized to exhibit laser, piezoelectric, ferroelectric, ferroelastic, and fluorescent properties for a long time. RE molybdates having general formula R_2_(MoO_4_)_3_ have been reported as a ferroelectric material [1]. The importance of these materials lies in their ferroelectric and ferroelastic properties finding immense application in electro- and acoustooptical devices [2–4]. RE heptamolybdate with general formula R_2_Mo_7_O_24, _belongs to the family of molybdates. Brixner [5] reported the growth of RE molybdates employing the Czochralski technique at elevated temperatures but the thermal stress introduced during the growth makes the crystal defective. In order to overcome these difficulties, it is worthwhile to grow some new RE molybdate crystals by other simple techniques. Large number of crystals has been grown at ambient temperature; therefore, growth at low temperature is expected to yield crystals with minimum defects. The crystal growth in gels at room temperature pioneered by Henisch and coworkers [6, 7] has been fully exploited in the investigation of growth and characterization of rare earth mixed crystals of Sm-Ba molybdate by Isac and Ittyachen [8]. Studies on the growth of pure lanthanum, neodymium, and mixed La-Nd heptamolybdate single crystals in silica gel have been carried out by Bhat et al. [9–13]. Growth, characterization, and thermal behaviour of pure Gd-heptamolybdate, mixed Gd-Ba molybdate, and Gd-Sr molybdate have also been carried out [14–16]. Crystallization and spherulitic growth of gadolinium, praseodymium, and mixed didymium heptamolybdate in silica gel medium [17, 18] have been investigated by many workers. Gadolinium molybdate (GMO) with general formula Gd_2_(MoO_4_)_3_ was observed to have luminescence properties [19]. Gofman et al. [19] studied radiation induced luminescence properties of gadolinium molybdate. Several other papers devoted to the luminescence properties of GMO doped with RE ions were also published [20–22]. Itoh [23] has studied luminescence properties of self-trapped exciton in CdMoO_4_. Keskar et al. [24] have carried out detailed study on thermal expansion of some other molybdates and tungstates such as Gd_2_Mo_3_O_12_ and Gd_2_W_3_O_12_. Korah et al. [25] reported the growth and structural characteristics of gadolinium neodymium oxalate crystals.

It is an established fact that an intrinsic material undergoes a change in its physical and chemical properties when it is grown in mixed form. Keeping in view the versatility of the gel technique in obtaining a large number of crystals, it was thought worthwhile to grow the mixed gadolinium calcium heptamolybdate (referred to as GdCaHM) crystal. In the present work, authors report the synthesis of GdCaHM crystals by controlled diffusion of chemical regents in silica gel medium. Literature survey reveals that no work has so far been reported on the synthesis and characterization of these crystals in sodium metasilicate gel at room temperature. Fourier transform infrared spectroscopy (FTIR) results suggest the presence of water of hydration in the grown crystal, so it was interesting to investigate in detail the thermal behaviour of these materials. Thus, this paper reports detailed results of synthesis, characterization, and thermal behavior of GdCaHM crystals grown by gel encapsulation technique.

## 2. Experimental Technique

### 2.1. Synthesis

The synthesis of gadolinium calcium heptamolybdate (GdCaHM) is accomplished by allowing controlled diffusion of Gd^3+^ and Ca^2+^ ions in equal ratio through silica gel impregnated with the lower reactant providing molybdenum ions. The growth of mixed GdCaHM is achieved by using the system (RCl_3_ + R^/^Cl_2_)–(NH_4_)_6 _Mo_7_O_24_–NH_4_NO_3_–HNO_3_–Na_2_SiO_3_ (where R = Gd^3+^ and R^/^ = Ca^2+^) by using single gel single tube technique. The crystallization apparatus used for the growth consists of borosilicate glass tube of 20 cm length and 2.5 cm diameter. The high quality pure grade chemicals, from S. D. fine Chemical Ltd., used are gadolinium chloride (99% A.R), calcium chloride (99% A.R), ammonium molybdate (99% A.R), ammonium nitrate (99% A.R), and sodium metasilicate (99% A.R).

In the present case, silica gel is used as it is optically transparent and therefore crystals can be observed during the growth experiment. Furthermore, unlike other gels, the use of silica gel minimizes the effect due to precipitate—precipitate interaction and crystal impact on the wall of the container. Therefore, silica hydrogel is preferred and is the more common gel used for the growth experiment. It is obtained by the neutralization of sodium metasilicate. The gelling reaction is based on the hydrolytic process given by the following chemical reaction:

(1) $$\begin{array}{c} \text{N}{\text{a}}_{2}\text{Si}{\text{O}}_{3}+3{\text{H}}_{2}\text{O}⟶{\text{H}}_{4}\text{Si}{\text{O}}_{4}+2\text{NaOH} \end{array}$$

Silica gel was prepared by dissolving 212.14 g of sodium metasilicate (Na_2_SiO_3_) in 1000 mL of distilled water so as to obtain gel solution of 1 M concentration. The sodium metasilicate solution was left undisturbed for few days and clear solution was obtained on decantation. The second solution of lower reactant was prepared by adding ammonium molybdate and ammonium nitrate each weighing 817 mg to 80 mL of distilled water. The solution was thoroughly mixed with the help of magnetic stirrer and then after about half an hour, 26 mL of concentrated HNO_3 _was added to it drop by drop till a white precipitate was formed. To this solution, 20 mL of water was added so as to make a total volume of 250 mL of lower reactant having 0.005 M concentration. The nitric acid content of the solution was added to adjust the desired pH value of the gel medium. The first solution (sodium metasilicate) was then mixed with the second (lower reactant—a source of molybdenum ions) in the crystallizer. The combined solution of desired pH was allowed to set in the crystallizer and then aged for desired time. Since in the present research work our objective is to grow mixed rare earth heptamolybdate crystal, therefore, upper reactant is a mixture of GdCl_3_ and CaCl_2_ in equal ratio. Upper reactant was prepared by dissolving 0.7 g of GdCl_3_ in 5 mL of H_2_O and 1.1 g of CaCl_2_ in 5 mL of H_2_O. The two solutions were then mixed to get the required solution of 0.5 M concentration in 1 : 1 ratio. This solution was then poured along the sides of the tube, ensuring that this process does not break the gel. The slow diffusion of upper reactant into the gel medium results into the reaction of rare earth/alkaline earth ion with heptamolybdate ion already present in the gel medium and subsequent formation of the proposed compound.

### 2.2. Characterization Technique

The different morphologies exhibited by the grown crystal were studied using an optical microscope (Epignost of Carl Zeiss Germany), whereas surface features of the grown crystals were studied using scanning electron microscopy (SEM, Model JEOL 840). The powder pattern was obtained using powder X-ray diffractometer (Rigaku Co. Ltd., Japan) with Cu K*α* radiation (*λ* = 1.54 Å) having scanning rate of 2°/min. Energy dispersive X-rays analysis (EDAX) was recorded using dispersive spectrometer (INCA ENERGY EDAX) attached to the scanning electron microscope for carrying out elemental analysis of the grown crystals. Fourier transform infrared (FTIR) spectra were recorded in the wave number range of 4000–500 cm^−1^ on the Perkin-Elmer 781 spectrophotometer using KBr pellet method. The thermal behavior was investigated using thermogravimetric analysis (TGA), differential thermal analysis (DTA), and differential scanning calorimetry (DSC). TGA and DTA curves were recorded simultaneously by a thermal analyzer (Shimadzu make DTG-60) over the temperature range from 25 to 1000°C in the N_2_ atmosphere at a heating rate of 10°C/min and flow rate of 30 mL/min. The DSC measurements were carried out on a DSC thermal analyzer (DSC-60 Shimadzu make) over a temperature range from 25 to 500°C in the nitrogen (N_2_) atmosphere at a heating rate of 10°C/min and flow rate of 30 mL/min.

## 3. Results and Discussion

### 3.1. Synthesis of Mixed Gd-Ca Heptamolybdate (GdCaHM)

In order to establish the optimum condition for the synthesis of GdCaHM in the form of single crystals of suitable size for scientific investigations, several experiments were performed under varying conditions of different growth parameters, namely, gel pH, gel concentration, concentration of upper and lower reactant, and gel ageing. The optimum conditions for the growth of mixed GdCaHM crystal is achieved by using the following parameters: molarity of gel (Na_2_SiO_3_): 0.5 M; molarity of lower reactant: 0.5 M; gel pH: 7.5; molarity of upper reactant: 0.5 M; and gelation period: 48 h.

### 3.2. Optical and Scanning Electron Microscopy

**Figure 1:**
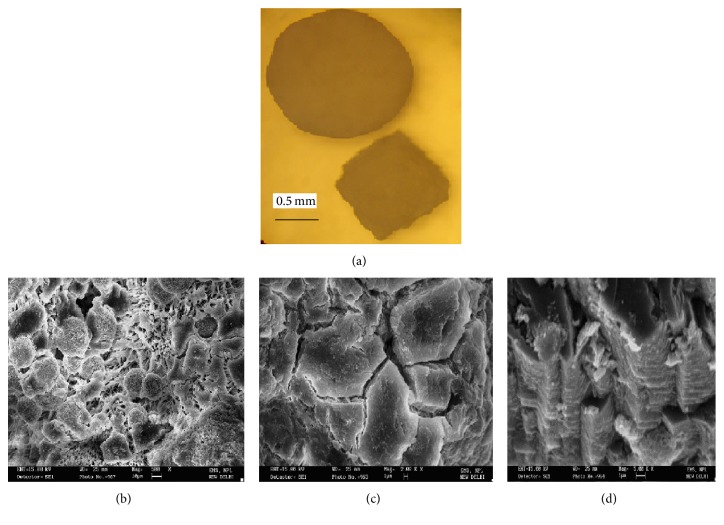
(a) Optical micrograph showing mixed Gd-Ca heptamolybdate (GdCaHM) crystal as a spherulite and a square platelet. (b) Scanning electron micrograph showing surface feature of spherulite crystals wherein number of spherulites are coalesced together with fiber like structure. (c) Scanning electron micrograph showing mixed GdCaHM crystal with cracks which results due to gold coating because of the presence of water content. (d) Scanning electron micrograph showing formation of growth layers in the growing crystal, suggesting two-dimensional nucleation and pilling up of growth layers.

Mixed Gd-Ca heptamolybdate (GdCaHM) crystals grown by single gel single tube technique assume various types of morphologies, which include spherulites, multifaceted, and square platelets.  Figure 1(a) is an optical micrograph showing the grown crystal as spherulite and square platelet. These spherulites are in fact an agglomeration of number of tiny crystals. In order to study the surface features, scanning electron microscopic study was performed on the grown crystal.  Figure 1(b) is an electron micrograph of GdCaHM showing surface features of the spherulite. Here, one can see number of small spherulites along with some fiber like structure. This fiber like structure can be due to the adhering of the silica gel. This adhering of the silica gel on some of the spherulite crystals is because of not being properly cleaned while taking crystal out of the crystallizer.  Figure 1(c) shows single crystal of GdCaHM with cracks. These cracks do not have specific crystallographic direction and results when the crystals were coated with gold in the vacuum coating plant and examined under scanning electron microscope. Such type of cracking is often associated with crystal having water of crystallization [13, 26, 27].  Figure 1(d) shows an interesting feature on one of the mixed Gd-Ca crystals. On close examination, it reveals growth layers suggesting two-dimensional spreading and piling up of growth layers, which is a consequence of preferred nucleation at the corners and at the edges of the face of growing crystal at relatively high supersaturation.

### 3.3. X-Ray Diffraction Analysis (XRD)

**Figure 2:**
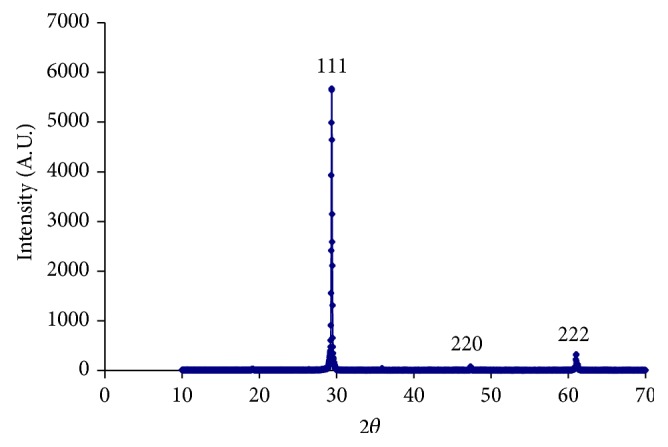
X-ray diffractogram of mixed GdCaHM showing various [hkl] planes.

The powder X-ray diffractogram of GdCaHM crystal is shown in Figure 2. The crystallinity is quite clear from diffractogram because of the occurrence of sharp peaks at specific 2*θ* Bragg angles. The details of the XRD plot depicting “*d*” spacing and corresponding [hkl] planes are given in Table 1.

### 3.4. Energy Dispersive X-Ray Analysis (EDAX)

**Figure 3:**
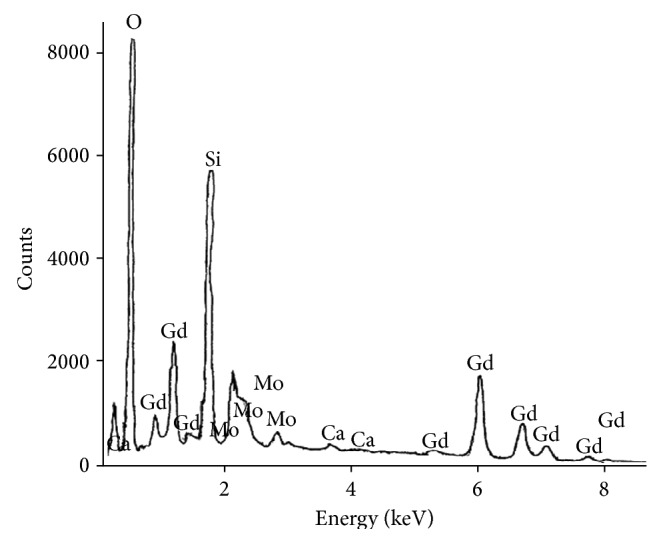
Energy dispersive X-ray analysis** (**EDAX) revealing the presence of major elements in the mixed GdCaHM crystal along with the presence of silica (Si).

To study the elemental composition, qualitative and quantitative analysis was performed by energy dispersive X-ray analysis (EDAX).  Figure 3 shows the EDAX pattern of the grown crystal. The EDAX pattern shows the peaks corresponding to Gd, Ca, Mo, O, and Si which suggest the presence of almost all the constituents expected to be present in the material. The presence of Si is attributed to the adhering of silica gel because of not being properly cleaned as has also been seen and discussed in Figure 1(b). Quantitative estimation of elements present in the crystal through its weight and atomic percentage based on the proposed composition GdCaMo_7_O_24_·8H_2_O is given in Table 2. The theoretical (quantitative) values based on the proposed composition are in good agreement with experimental (qualitative) values; thus, based on this analysis, it is clear that these crystals belong to the heptamolybdate series with the composition GdCaMo_7_O_24_·8H_2_O. The water of hydration associated with this system is revealed by FTIR spectrum as given in the next section. The proposed composition was further verified by the thermal analysis given in Section 3.6 (TGA, DTG, and DTA) which support the inference that the material is associated with water of hydration, which dissociates from the material at different stages of decomposition occurring at different temperature intervals.

### 3.5. Fourier Transform Infrared Spectroscopy (FTIR) 

**Figure 4:**
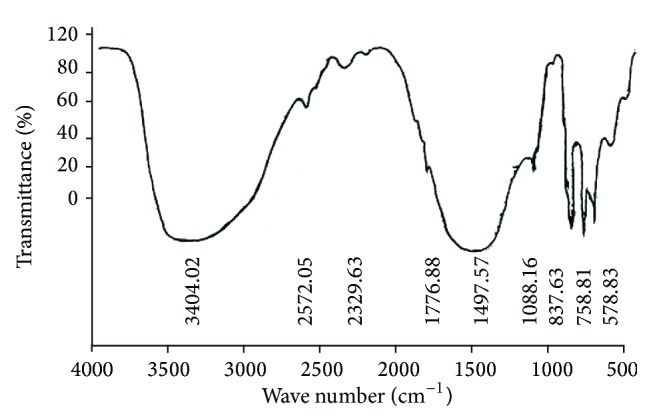
FTIR spectrum of the crystal depicting the various functional groups present in the mixed GdCaHM crystal.

Figure 4 shows an infrared spectrum for GdCaHM crystals recorded by using KBr pellet technique, for wave numbers in the range from 500 to 4000 cm^−1^. As seen from the spectrum, there is a broad and strong peak at 3404.2 cm^−1^ which is due to water and strong stretching modes of the OH^−^ group (water symmetry structure). The weak and broad peak observed at 2329.63 cm^−1^ can be attributed to the presence of Si–H bond. EDAX analysis also confirms the presence of Si in the material. The strong and broad peak at 1497.57 cm^−1^ is indicative of vibration of oxygen ion for Gd. The strong, sharp peak observed at 837.63 cm^−1^ is due to symmetric or antisymmetric stretching vibrations of the heptamolybdate ion (Mo_7_O_24_) [28–30]. The peaks occurring at 758.81 and 578.83 cm^−1^ are attributed to the presence of metal oxygen bonds. A comparative assignment of prominent peaks of FTIR spectra is given in the Table 3.

### 3.6. Thermal Analysis

**Figure 5:**
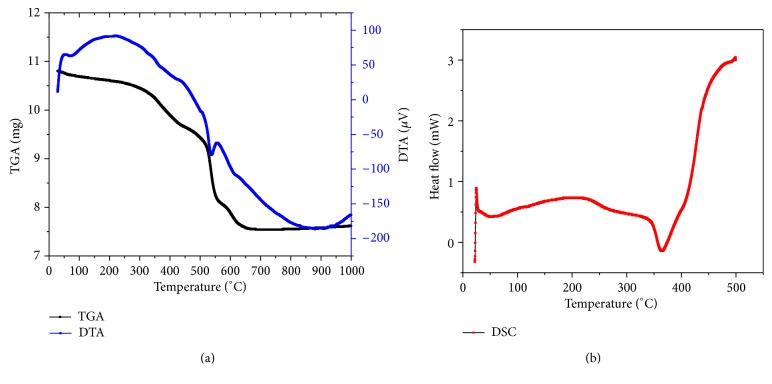
(a) Thermal analysis curve showing simultaneous recording of TGA, DTG, and DTA curves for mixed GdCaHM crystal. (b) DSC plot of mixed GdCaHM crystal.

Thermal analysis is a very useful technique in order to assess the thermal stability of a material. Thermal behavior of GdCaHM has been studied by using various thermoanalytical techniques which include thermogravimetry (TG), differential thermal analysis (DTA), and differential scanning calorimetry (DSC).  Figure 5(a) shows the thermograph where in TG and DTA curves are simultaneously recorded. The recorded thermograph (Figure 5(a)) was first analyzed to obtain information about the percentage of mass loss at different temperatures and hence about crystal stability. From the curve, it is clear that when the crystal is heated at a uniform rate of 10°C min^−1^, its mass is found to be lost continuously as a function of applied temperature. The thermal decomposition takes place in three different stages starting from 28 to 666°C.

The first stage of decomposition begins at a temperature of 28°C and get completed at 440°C, leading to weight loss of 10.38% (Table 4). The weight loss in the first stage of decomposition is attributed to the loss of eight water (H_2_O) molecules from the grown composition. The calculated loss of eight water molecules (8H_2_O) comes out to be 10.31% which is close approximation to the observed values, thereby confirming the results from EDAX analysis that the composition consists of eight water molecules with it. The second stage of decomposition starts at 441°C which continuous up to 543°C. Up to the end of this stage, a weight loss of 11.49% is observed. The weight loss in the second stage corresponds to the removal of one molybdenum (MoO_3_) molecule. The third decomposition stage begins at 544°C and continuous up to 666°C leading to weight loss of 6.49%. The weight loss in the third stage is due to the loss of one calcium peroxide (CaO_2_) molecule. The end product obtained after the third stage of decomposition is anhydrous gadolinium hexamolybdate (GdMo_6_O_19_).

From the TG curve, which is reported up to a temperature of 1000°C, it is clear that material remains stable after the third stage of decomposition is completed. Table 4 gives the summary of results obtained from the thermal decomposition for different temperature ranges with composition as GdCaMo_7_O_24_·8H_2_O. It can be seen that the calculated weight losses are in close proximity with the observed values. DTA curve shows an exothermic peak at 225°C, which corresponds to the first stage of decomposition of TG curve. There is well-marked endothermic peak in DTA curve at 535°C, which corresponds to the second stage of decomposition of TG curve. The third peak (exothermic) occurs at 565°C, which corresponds to the third stage of decomposition of TG curve.  Figure 5(b) is a representative DSC plot of GdCaHM crystals. From the DSC plot, one can see an endothermic peak to which corresponding enthalpy change is about 19.97 mJ. The second endothermic peak in the DSC plot is at a temperature of 365°C thereby suggesting that there is a mass loss in the material which is in quite good agreement with the mass loss in the TGA curve. The enthalpy involved in this endothermic peak is −96.68 mJ.

### 3.7. Solid State Kinetic Parameters of Thermal Decomposition Analysis

For studying the kinetics of solid state decomposition, three equations, namely, Horowitz-Metzger [31], Coats-Redfern [32], and Piloyan-Novikova [33], were used for calculating various kinetic parameters such as activation energy (*E*_*a*_), order of reaction (*n*), frequency factor (*Z*), and entropy (Δ*S* ^*^). The kinetics was studied by nonisothermal methods in the temperature range of 28–666°C. As the decomposition in the constituent element takes place in three different stages (Figure 5(a)), so all the three stages of decomposition were used for calculating various kinetic parameters.

**Figure 6:**
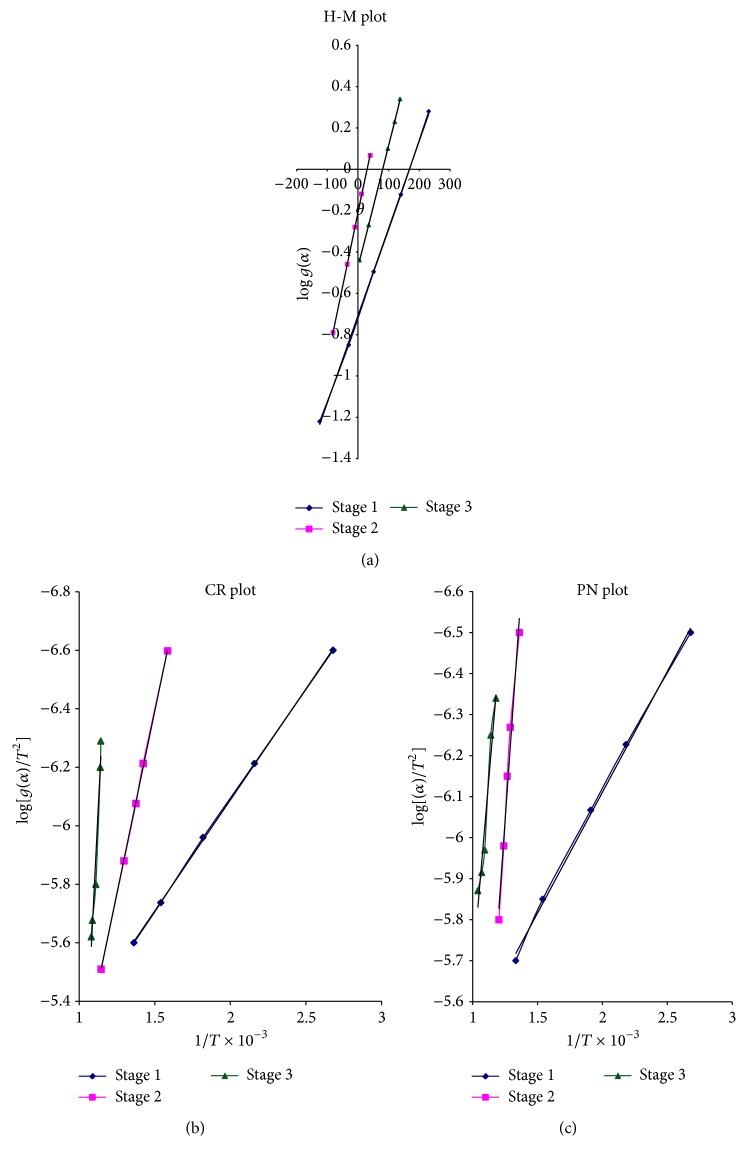
(a) Horowitz-Metzger plot of GdCaHM for three stages of decomposition. (b) Coats-Redfern plot of GdCaHM for three stages of decomposition. (c) Piloyan-Novikova plot of GdCaHM for three stages of decomposition.

In case of Horowitz-Metzger relation [31], a graph was plotted between log⁡⁡[*g*(*α*)] versus *θ* for different values of *n* (order of reaction) = 1, 1/2, 1/3, 2/3.  Figure 6(a) shows the best linear fit for *n* = 1/2, that is, for *g*(*α*) = 2[1(1−*α*)^1/2^]. Since the value of order of reaction “*n*” comes out to be 1/2, thereby suggesting that the mechanism of decomposition is based on contracting cylindrical model. In this model, the nucleation occurs rapidly on the decomposing nuclei and the rate of degradation is controlled by the resulting reaction interface, which progress towards the center of the crystal. The derivation of the mathematical model for contracting cylindrical decomposition depends on the crystal shape. This decomposition model has also been observed in case of pure and cadmium doped barium phosphate single crystals [34]. Dehydration of calcium oxalate monohydrate also follows geometrical contraction model [35]. The activation energy (*E*_*a*_) was calculated from the slope of the graph between log⁡⁡[*g*(*α*)] versus *θ* which for first stage comes out to be 22.3 KJmol^−1^. The value of the frequency factor (*Z*) and entropy (Δ*S* ^*^) was also calculated using the various relations [34] which comes out to be 1.19 S^−1^ and −247 JK^−1^ mol^−1^, respectively, for first stage of decomposition. Similarly, it has been observed that on applying Horowitz-Metzger relation, the value of order of reaction (*n*) comes out to be 1/2 for second and third stage of decomposition thereby suggesting that for all the three stages of decomposition contracting cylindrical model is applicable.  Figure 6(a) shows the best linear fit for all the three stages of decomposition. The energy of activation (*E*_*a*_), frequency factor (*Z*), and entropy (Δ*S* ^*^) were also calculated for second and third stages of decomposition, the values of which are given in Table 5.

Coats-Redfern relation [32] was also applied to find the mechanism of decomposition for three different stages.  Figure 6(b) shows graph of log⁡⁡[*g*(*α*)/*T* ^2^] versus 1/*T* for *n* = 1/2, where *g*(*α*) = 2[1(1−*α*)^1/2^]. The order of reaction (*n*) from Coats-Redfern relation comes out to be 1/2, which again confirms that decomposition proceeds according to contracting cylindrical model as has been observed in case of Horowitz-Metzger relation. The energy of activation (*E*_*a*_), frequency factor (*Z*), and entropy (Δ*S* ^*^) were calculated using appropriate equations for all the three stages of decomposition and the values are shown in Table 5.

Piloyan-Novikova relation [33] does not give the value of order of reaction and hence no model for decomposition. However, from this relation by plotting a graph between log⁡⁡[(*α*)/*T* ^2^] versus 1/*T*, one can calculate the value of activation energy (*E*_*a*_) and frequency factor (*Z*) from the slope and intercept, respectively.

Table 5 thus gives compiled data for various kinetic parameters, that is, energy of activation (*E*_*a*_), order of reaction (*n*), frequency factor (*Z*), and entropy (Δ*S* ^*^) obtained on applying the equations of Horowitz-Metzger, Coat-Redfern, and Piloyan-Novikova relations for mixed GdCaHM crystal. From these kinetic parameters and from all the three different stages of decomposition, we conclude that in case of mixed GdCaHM crystal, the maximum activation energy (*E*_*a*_) is required for second stage of decomposition because in this stage molybdenum trioxide (MoO_3_) decomposes from the system whereas minimum activation energy is required for the first stage of decomposition because in this stage water (H_2_O) is decomposed.

## 4. Conclusions

From the results obtained on mixed gadolinium calcium heptamolybdate (GdCaHM) crystals one can draw the following broad conclusions.

(i) Crystals of GdCaHM having composition GdCaMo_7_O_24_·8H_2_O have been successfully synthesized by single gel single tube technique.
(ii) The optical and scanning electron microscopic studies reveal growth of mixed GdCaHM crystals, which exhibit various morphologies. Thus, mixed GdCaHM crystal grows as single crystal with square platelet as well as spherulites.
(iii) The qualitative and quantitative elemental analysis, employing energy dispersive X-ray analysis, confirms the growth of mixed GdCaHM crystals. The calculated and experimental weight percentage for different elements is in good agreement for the composition GdCaMo_7_O_24_·8H_2_O.
(iv) The FTIR spectroscopy establishes the crystal to be hydrated with all functional groups expected to be present in the crystal.
(v) GdCaHM crystals are stable up to a temperature of 28°C and then starts decomposing and this decomposition process continues up to 666°C. The whole decomposition process is completed in three different stages of decomposition.
(vi) DTA and DSC curves indicate that there may be some physical transformations (besides mass changes associated with loss of water) in case of mixed GdCaHM crystal.
(vii) By using Horowitz-Metzger and Coats-Redfern equations for all the three different stages of decomposition, the best linear fit is obtained for an order of reaction *n* = 1/2. This indicates that the decomposition proceeds according to the contracting cylindrical kinetic model in GdCaHM crystal.
(viii) The various kinetic parameters like energy of activation (*E*_*a*_), frequency factor (*Z*), and entropy of decomposition (Δ*S* ^*^) using three different equations were calculated for all the three stages of decomposition, indicating that minimum activation energy is required to break the molecules in the first stage of decomposition because the water molecules are loosely bound to the mixed crystal.

## Figures and Tables

**Table 1:** Compiled data of various hkl planes corresponding to different Bragg angle and inter planer spacing for mixed GdCa heptamolybdate (GdCaHM).

*d*-spacing (Å)	2 theta (*θ*)	[hkl] planes	Intensity (counts)
3.14	29.38	111	6173
2.10	47.46	220	559
1.77	61.04	222	**823**

**Table 2:** Experimental and theoretical calculated composition obtained from energy dispersive X-ray analysis (EDAX) of various constituent elements present in case of GdCa heptamolybdate (GdCaHM) crystal for composition of GdCaMo_7_O_24_·8H_2_O.

Element	Experimental values		Theoretical values	
	Wt. (%)	At. (%)	Wt. (%)	At. (%)
Oxygen	36.87	77.57	37.06	78.04
Calcium	3.38	2.71	2.9	2.43
Molybdenum	47.5	17	48.65	17.07
Gadolinium	12.25	2.71	11.3	2.43

**Table 3:** Infrared band/peak assignments of some selected bands/peaks for mixed GdCa heptamolybdate (GdCaHM) crystal.

IR band/cm^−1^	Assignments of bands/peaks
3404.2	Water symmetry structure
2329.63	Presence of Si–H bond
1776.63	Attributed to water bending
1497.57	Vibration of oxygen ion for gadolinium
837.63	Vibrations of molybdate ions (Mo_7_O_24_)
578.83	Presence of metal oxygen bond

**Table 4:** Results of thermal decomposition for different temperature ranges with observed and calculated weight loss for mixed crystal with composition GdCaMo_7_O_24_·8H_2_O.

Stage	Temp. (°C)	Decomposition step	Weight loss (%)	
			Obsv.	Cal.
Ist	28–440	GdCaMo_7_O_24_·8H_2_O → GdCaMo_7_O_24_ + 8H_2_O	10.38	10.31
IInd	441–543	GdCaMo_7_O_24_ → GdCaMo_6_O_21_ + MoO_3_	11.41	11.49
IIIrd	544–666	GdCaMo_6_O_21_ → GdMo_6_O_19_ + CaO_2_	7.77	6.49

**Table 5:** Various kinetic parameters calculated from the TGA using three different relations.

STAGE	TG method	Order of reaction (*n*)	Activation energy (K Jmol^−1^)	Frequency factor *Z* (S^−1^)	Entropy Δ*S* ^*^ (JK^−1^mol^−1^)
First	Horowitz-Metzger	*n* = 1/2	22.3	1.19	−247
	Coats-Redfern	*n* = 1/2	25.7	2 × 10^−4^	−259.75
	Piloyan-Novikova	nil	9.514	4.4 × 10	−294.3

Second	Horowitz-Metzger	*n* = 1/2	152.31	1.0 × 10^8^	−99.46
	Coats-Redfern	*n* = 1/2	111.86	1.6 × 10^5^	−153.11
	Piloyan-Novikova	nil	89.22	1.1 × 10	−328.68

Third	Horowitz-Metzger	*n* = 1/2	100.85	1.6 × 10^4^	−172.67
	Coats-Redfern	*n* = 1/2	87.05	2.3 × 10^−5^	−341.97
	Piloyan-Novikova	nil	38.12	9 × 10^−2^	−273.53
